# Effects of Megafol on the Olive Cultivar ‘Arbequina’ Grown Under Severe Saline Stress in Terms of Physiological Traits, Oxidative Stress, Antioxidant Defenses, and Cytosolic Ca^2+^

**DOI:** 10.3389/fpls.2020.603576

**Published:** 2021-01-15

**Authors:** Daniele Del Buono, Luca Regni, Alberto Marco Del Pino, Maria Luce Bartucca, Carlo Alberto Palmerini, Primo Proietti

**Affiliations:** Department of Agricultural, Food and Environmental Sciences, University of Perugia, Perugia, Italy

**Keywords:** plant biostimulant, photosynthesis, salt stress, oxidative stress, antioxidant enzymes, Ca^2+^ homeostasis

## Abstract

Salinity is one of the most impacting abiotic stresses regarding crop productivity and quality. Among the strategies that are attracting attention in the protection of crops from abiotic stresses, there is the use of plant biostimulants. In this study, Megafol (Meg), a commercial plant biostimulant, was tested on olive plants subjected to severe saline stress. Plants treated with salt alone showed substantial reductions in biomass production, leaf net photosynthesis (Pn), leaf transpiration rate (E), stomatal conductance (gs), and relative water content (RWC). In addition, samples stressed with NaCl showed a higher sodium (Na^+^) content in the leaves, while those stressed with NaCl and biostimulated with Meg increased the potassium (K^+^) content in the leaves, thus showing a higher K^+^/Na^+^ ratio. Salinity caused the accumulation of significant quantities of hydrogen peroxide (H_2_O_2_) and malondialdehyde (MDA) due to decreases in the activity of antioxidant enzymes, namely superoxide dismutase (SOD – EC 1.15.1.1), ascorbate peroxidase (APX – EC 1.11.1.11), guaiacol peroxidase (GPX – EC 1.11.1.9), and catalase (CAT – EC 1.11.1.6). When olive plants under saline stress were biostimulated with Meg, the plants recovered and showed physiological and biochemical traits much improved than salt stressed samples. Finally, Meg exhibited Ca^2+^-chelating activity in olive pollen grains, which allowed the biostimulant to exert this beneficial effect also by antagonizing the undesirable effects of hydrogen peroxide on Ca^2+^ metabolism.

## Introduction

Abiotic stresses can strongly affect plant growth and development and impact crop quality and productivity, primarily if they occur during the most sensitive phenological phases (Bulgari et al., 2019). Salinity is among the stresses with the highest impacts on agriculture due to its highly detrimental effect on crops (Naifer et al., 2011). The use of saline water for irrigation and the consequent accumulation of salts in soils have worsened the situation over the past 20 years (Acosta-Motos et al., 2017), and it has been estimated that, currently, salinity affects about 800 million hectares of arable land all around the world (Acosta-Motos et al., 2017).

Salinity can induce a variety of physiological, morphological, and biochemical changes in plants, affecting plant establishment and causing stunted growth (Lucini et al., 2015). These alterations are due to osmotic and ionic effects, which hamper adequate nutrient acquisition and translocation, reduce enzyme activities and interfere with pivotal metabolic processes such as photosynthesis (Pn; Colantoni et al., 2017). Excessive salt uptake can also interrupt or hinder the production of specific metabolites which directly regulate plant growth (Rouphael et al., 2018). Salt injuries can also provoke the death of leaves because of elevated saline levels in the cell wall or cytoplasm as a result of the incapability of the vacuole to sequester salt over a longer period (Munns, 1992). Saline stress can also interfere with the transfer of carbohydrates and hormones to meristematic regions (Munns, 1992) and may result in an increased root-to-shoot ratio and in altered root morphology (Hsiao and Xu, 2000; Acosta-Motos et al., 2015). Other effects regard the reduction of the total leaf area and, subsequently, of the canopy, although this is thought to be a plant response to stress to minimize water loss by transpiration (Ruiz-Sánchez et al., 2000). Despite this, some plants have developed mechanisms to reduce or avoid injuries caused by salinity. For instance, stomatal closure has often been observed to minimize water loss by transpiration (Alarcón et al., 2006). However, stomatal closure declines photosynthetic activity, which in turn decreases CO_2_ assimilation (Alarcón et al., 2006). Decreases in stomatal conductance, imposed by saline stress, reduce the CO_2_:O_2_ ratio and increase photorespiration to prevent photo-oxidative damage (Kangasjärvi et al., 2012).

Plants tolerance to salinity, being often dependent on the species and/or cultivar, has been linked, in some cases, to mechanisms which limit the import of Na^+^ at the shoot level (Chartzoulakis et al., 2002). This protective action occurs in the roots level and reduces or prevents salt translocation (Chartzoulakis et al., 2002; Mousavi et al., 2019). Nonetheless, this mechanism seems to be effective at low NaCl concentrations up to 50 mM, while, at higher salinity, Na^+^ in most species can accumulate in the aerial parts, thus causing toxicity (Chartzoulakis et al., 2002). Furthermore, the plant tolerance to salt stress may also depend on its ability to increase K^+^ levels in the leaves (Mousavi et al., 2019). In particular, the accumulation of a solute, such as K^+^, determines osmotic adjustment, which increases plant tolerance to salinity (Elansary et al., 2017; Del Buono, 2021).

There is plenty of literature documenting that salinity gives rise to oxidative stress, mainly in chloroplasts and mitochondria, causing overproduction of reactive oxygen species (ROS; López-Gómez et al., 2007; Acosta-Motos et al., 2017; Elansary et al., 2017; El Arroussi et al., 2018; Del Buono, 2021). The coordinated upregulation of the antioxidant system is a potential response activated by species to cope with oxidative perturbations. Salt-tolerant species induce antioxidant enzyme production, thus improving their capacity to remove ROS (López-Gómez et al., 2007). In contrast, salt-sensitive species display decreases in antioxidant activity (López-Gómez et al., 2007). The main enzymes activated by plants to cope with abiotic oxidative stress are superoxide dismutase (SOD), ascorbate peroxidase (APX), guaiacol peroxidase (GPX), and catalases (CAT; Lee et al., 2013; Proietti et al., 2013; Ikbal et al., 2014; Mimmo et al., 2015; Del Buono et al., 2016; Panfili et al., 2019a). SOD acts by disproportioning O_2_^−^ to O_2_ and H_2_O_2_ (Mittova et al., 2002; Ikbal et al., 2014); the resulting H_2_O_2_ is scavenged by APX, GPX, and CAT (Mittova et al., 2002; Ikbal et al., 2014).

Calcium (Ca^2+^) is an essential nutrient in plants, with concentrations ranging from 0.1% to over 5% of dry weight (White and Broadley, 2003). It, therefore, has a dual function, both as a structural component of the walls and cell membranes and a second intracellular messenger. Therefore, absorption, distribution, and storage must be finely regulated to satisfy both functions (Thor, 2019). In addition to its structural role, Ca^2+^ is a second messenger in a variety of processes affecting the growth and fertilization of the pollen tube in responses to abiotic and biotic stresses, including drought and salinity (Knight et al., 1997; Lecourieux et al., 2005; Michard et al., 2009; Dodd et al., 2010; Monshausen et al., 2011; Steinhorst and Kudla, 2013; Ortiz-Ramírez et al., 2017). The transient increases in cytosolic Ca^2+^, hence, serve as a signal to trigger downstream responses (Dodd et al., 2010; Thor and Peiter, 2014). To activate this function, the levels of cytosolic Ca^2+^ in unstimulated conditions must be maintained at concentrations below 0.1 μM (Dodd et al., 2010; Kudla et al., 2018).

Olive generally shows a moderate capacity to tolerate salinity (Perica et al., 2008). Recently, olives have been cultivated with low-quality water (high salt content) in arid or semi-arid areas (Perica et al., 2008). Furthermore, to exacerbate this situation, olive cultivation progressively extended to areas where salinity has become a significant issue due to the high evapotranspiration and insufficient soil leaching (Chartzoulakis, 2005). In olive, salinity can cause decreased Pn rates, chlorophyll content, and plant growth, as well as increased oxidative stress (Regni et al., 2019). The cv. ‘Arbequina’, reported in literature as medium tolerant to salt stress (Weissbein et al., 2008), was chosen for the experiment since its use in the world is rapidly increasing. In fact, this cultivar proved its adaptability to new high-density olive planting (Caruso et al., 2014). The ‘Arbequina’ cv. is having the best results in super high-density olive groves, which, in recent years, have an increase in interest in all the olive growing areas in the world. The good adaptability of ‘Arbequina’ cv. to super high-density systems is due to low vigor, high branching density, and high fruit-bearing capacity (Rosati et al., 2018a,b) which is a high assimilate demanding process (Famiani et al., 2019).

Among the promising strategies adopted to improve plant resistance to abiotic stress, the use of plant biostimulants in agriculture has recently been proposed (Panfili et al., 2019b; Almadi et al., 2020). Biostimulants enhance plant nutrient uptake and use efficiency, and there is evidence that they increase plant resistance to stress (Calvo et al., 2014). They are grouped based on their origin: plants, seaweeds, animals, bacteria, fungi, and raw materials containing humates (Yakhin et al., 2017). The biological function of a biostimulant is to exert a beneficial effect on plants, although an explicit mode of action has not been identified (Yakhin et al., 2017). The complexity and the multicomponent nature of biostimulants make it difficult to determine their mode of action. Yet, a biostimulant should be considered solely for its demonstrated positive impact on crops (Yakhin et al., 2017).

To date, the use of biostimulants in improving olive resistance to salinity has not been investigated, despite the negative consequences of this stress on olive production (Regni et al., 2019). For the reasons mentioned above, we determined the effect of Megafol (Meg), a commercial plant biostimulant (Panfili et al., 2019b) registered for the use in olive cultivation. In particular, we investigated the impact of salinity on olive grown in hydroponic solutions and the action of Meg in regard to salt stress. The physiological, morphological, and biochemical responses of plants subjected to salinity and biostimulated with Meg were compared with those exhibited by un-biostimulated plants grown in saline conditions and control samples. Finally, the biomolecular effects of Meg on cytosolic Ca^2+^ homeostasis were evaluated. As an experimental model, we used olive pollen grains in basal conditions and *in vitro*, inducing oxidative stress with hydrogen peroxide.

## Materials and Methods

### Plant Material, Treatments, and Growing Conditions

Rooted cuttings (15 cm average height) of olive plants, cv. ‘Arbequina’, grown in hydroponic condition, were used. Compared to growing in a normal substrate, the hydroponic system allows better control of factors such as temperature and humidity. Immediately before placing the plants in the hydroponic system, they were removed from the perlite of the mist propagation system and, after root washing with distilled water, placed in 800 ml pots containing expanded clay and kept under hydroponic conditions for an adaptation period of 60 days. The recirculating hydroponic solution was composed of half-strength Hoagland solution (pH 7.5), and the hydroponic system consisted of PVC containers. Each container, including five plastic hydroponic pots, was connected to a tank (volume 3.5 L each) containing the nutrient solution. An automated system ensured the flux of the nutrient solution from the tank to the PVC containers three times per day, and the nutrient solution was replaced once a week.

The hydroponic system was maintained in a growing chamber, and plants were exposed to light with a photosynthetic active radiance by a system equipped with lamps (PHILIPS SON-T AGRO 400 W) producing 200 μmol m^−2^ s^−1^ photon flux density, under a photoperiod of 16 h d^−1^. The temperature was constant at 23°C (+/−1°C), at a relative humidity of about 60%.

At the end of the adaptation period to hydroponic conditions (60 days), 30 plants were subjected to salt stress by adding 150 mM NaCl to the solution, while control plants continued to grow in NaCl-free nutrient solution. Fifteen stressed plants where treated two times (at 7 and 14 days after the beginning of salt stress), with 150 ml of the biostimulant Meg at a concentration of 2.5 ml L^−1^. A decrease in Pn was observed in stressed trees compared to the control starting at 7 days from the beginning of salt stress, which resulted in treatment with Meg.

Therefore, the treatments in the experiment were control plants, salt-stressed plants (NaCl), and salt-stressed plants plus the biostimulant treatment (NaCl + Meg) with three replicates (3 containers x 5 plants = 15 plants) for each treatment.

### Plant Status and Growth

During the experiment, visible stress symptoms (e.g., leaf alteration, plant death, etc.) were evaluated once a week. At the end of the experiment, 40 days after the beginning of NaCl treatment (DAT), six plants from each treatment were selected, and roots, shoots, stems, and leaves of each plant were weighed fresh (FW) and then oven-dried at 95°C until constant weight to determine dry weight (DW).

### Leaf Gas Exchanges, Relative Water Content, and Chlorophyll Content

Leaf net Pn, leaf transpiration rate (E), stomatal conductance (gs), sub-stomatal CO_2_ concentration (Ci), and relative water content (RWC) were determined for each treatment at 7, 14, and 21 DAT. Leaf gas exchange rates were measured using a portable IRGA (ADC-LCA-3, Analytical Development, Hoddesdon, UK) and a Parkinson-type assimilation chamber. Leaves were enclosed in the chamber and exposed to the same light as in the hydroponic system. The flow rate of air passing through the chamber was kept at 5 cm^3^ s^−1^. During gas-exchange measurements, the external CO_2_ concentration was about 375 cm^3^ m^−3^, and the air temperature inside the leaf chamber was about 1°C higher than the hydroponic room temperature. Measurements were taken under steady-state conditions (after about 30 s); Pn, gs, and E were expressed on a leaf-area basis.

The relative water content was calculated as follows:

$$\mathrm{RWC}\;\left(\%\right)=\left[\left(\mathrm{FW}-\mathrm{DW}\right)/\left(\mathrm{TW}-\mathrm{DW}\right)\right]\;x\;100,$$

where FW is the fresh weight, DW is the dry weight, and TW is the turgid weight of leaves.

After determining fresh leaf weight, the leaves were placed in distilled water for 24 h at room temperature (about 15°C) under dark conditions. Subsequently, after drying the leaf surface with absorbent paper towels, the turgid weight (TW) was recorded. Leaves were then oven-dried at about 85°C to constant weight (DW).

The chlorophyll content was measured on 15 leaves for each treatment, using a SPAD-502 Chlorophyll Meter (Minolta Camera Co. Ltd., Japan) at 7, 14, and 21 DAT.

### Na^+^, K^+^, Hydrogen Peroxide, and Malondialdehyde Contents in Leaves

The Na^+^ and K^+^ content was determined in olive leaves harvested at 40 days after the treatment with NaCl. For this purpose, control samples and plants treated with NaCl, in combination or not with Meg, were collected, dried at 70°C for 48 h (until a constant weight was reached), grounded, added with HNO_3_ 65% (v/v) and H_2_O_2_ 30% (v/v), and digested at 90°C. The concentration of Na^+^ and K^+^ in the acid digested leaves was determined using a flame photometer.

The contents of H_2_O_2_ and MDA were determined in olive leaf samples at 21 days after Meg treatment. H_2_O_2_ was quantified according to Velikova et al. (2000) in control samples and NaCl-stressed plants treated with or without Meg. Olive leaves (0.5 g FW) were homogenized in trichloroacetic acid (TCA) 0.1% (w/v) using a mortar and pestle, adding small amounts of quartz sand, followed by centrifugation at 12,000 *g* for 15 min. Then, 0.5 ml of the supernatant was transferred into a plastic cuvette containing a solution composed of 0.5 ml of 10 mM KH_2_PO_4_/K_2_HPO_4_ (pH 7.0) and 1.0 ml of 1 M KI. The mixture was then kept at room temperature in the dark, and after 10 min of incubation, absorbance was determined spectrophotometrically at 390 nm. The H_2_O_2_ concentration in the samples was estimated using a calibration curve (Velikova et al., 2000).

To determine the malondialdehyde (MDA) content, olive leaves (0.2 g FW) were extracted in a solution containing 10% (w/v) trichloroacetic acid and 0.25% (w/v) thiobarbituric acid and centrifuged for 20 min at 10,000 *g* (Panfili et al., 2019a); the obtained supernatant was kept in a water bath (95°C) for 30 min. After rapid cooling, absorbance was determined spectrophotometrically at 532 and 600 nm (Panfili et al., 2019a).

### Enzyme Extraction and Activity Determination

The activity of some antioxidant enzymes was determined in control samples, NaCl-stressed plants treated with or without Meg, at 21 DAT. Olive leaves (0.5 g FW) were ground in liquid nitrogen, adding small amounts of quartz sand, and subjected to different extractions, depending on the enzyme to assay. In particular, SOD, GPX and CAT samples were extracted (1:5 w/v) in 50 mM KH_2_PO_4_/K_2_HPO_4_ (pH 7.8). Regarding the APX enzyme, samples were extracted in a solution (1:5 w/v) containing 0.1 M Tris (pH 7.5), 2 mM ethylenediaminetetraacetic acid (EDTA), 1 mM dithiothreitol (DTT), and 1.5% polyvinylpolypyrrolidone (PVPP).

The enzyme extracts were then filtered through Miracloth and centrifuged for 15 min at 15,000 *g* (4°C). Total protein in the extracts was determined according to Bradford (1976).

### Enzyme Assays

For the SOD assay, 2.60 ml of 50 mM KH_2_PO_4_/K_2_HPO_4_ (pH 7.8), containing 0.1 mM EDTA and 13 mM L-methionine, was placed into a plastic cuvette and spiked with 50 μl of the enzymatic extract (2.6.1), 300 μl of 75 μM nitro blue tetrazolium (NBT), and 30 μl of 2 μM riboflavin. Samples were then exposed to fluorescent lamps (15 W) for 15 min at 25°C, and the photoreduction of NBT was recorded as the increase in absorbance (560 nm). The non-enzymatic reaction was carried out without enzyme extract. One unit of SOD was calculated as the amount of enzyme causing 50% inhibition of NBT reduction compared to the blank, according to Beyer and Fridovich (1987).

To determine APX activity, 100 μl of plant extract (2.6.1) was placed into a quartz cuvette and spiked of 2.0 ml of 50 mM KH_2_PO_4_/K_2_HPO_4_ (pH 7.0), 100 μl of 0.05 mM ascorbic acid, and 25 μl of 30% H_2_O_2_. The reaction was monitored spectrophotometrically for 1 min at 290 nm, and APX activity was determined according to Nakano and Asada (1981).

For the GPX assay, 850 μl of 40 mM KH_2_PO_4_/K_2_HPO_4_ (pH 7.0) containing 0.1 mM EDTA was spiked with 50 μl of enzymatic extract (2.6.1), 50 μl of 100 mM guaiacol, and 50 μl of 0.3 mM H_2_O_2_. The increase in absorbance due to guaiacol oxidation was monitored spectrophotometrically for 1 min at 470 nm; GPX activity was determined according to Upadhyaya et al. (1985).

Catalase activity was determined according to Aebi (1984). Specifically, 2 ml of 50 mM KH_2_PO_4_/K_2_HPO_4_ (pH 7.8) buffer was placed in a quartz cuvette and spiked with 100 μl of enzymatic extract (2.X.1) and 0.5 ml of 200 mM H_2_O_2_; CAT activity was determined spectrophotometrically for 1 min at 290 nm (Aebi, 1984).

### Determination of Cytosolic Ca^2+^ in Olive Pollen

Cytosolic Ca^2+^ levels were determined spectrofluorometrically using the probe FURA-2 AM (Del Pino et al., 2019a,b). Olive pollen (100 mg) from sub-samples stored in the dark at 5°C was suspended in 10 ml PBS and hydrated for 2 days at 25°C. Hydrated pollens were harvested by centrifugation at 1,000 *g* × 4 min and then resuspended in 2 ml HBSS buffer (120 mM NaCl, 5.0 mM KCl, 1 mM MgCl_2_, 2 mM CaCl_2_, 5 mM glucose, 25 mM Hepes, pH 7.4). Pollen suspensions were incubated in the dark with FURA-2 (2 μl of a 2-mM solution in DMSO) for 120 min, followed by centrifugation at 1,000 *g* × 4 min. Pollens were then harvested and suspended in ~10 ml of HBSS containing 0.1 mM EGTA, which was included to rule out or, at least, minimize a potential background due to contaminating ions (to obtain a suspension of 1 × 10^6^ of pollen granules hydrated per ml). Fluorescence was measured in a Perkin-Elmer LS 50 B spectrofluorometer (ex. 340 and 380 nm, em. 510 nm), set with a 10-nm and a 7.5-nm slit width in the excitation and emission windows, respectively. Fluorometric readings were taken after 300–350 s. When required, samples of pollen, H_2_O_2_ and Meg were added for specific purposes, as described in the Results section. Cytosolic calcium concentrations [(Ca^2+^)_c_] were calculated as described in Grynkiewicz et al. (1985).

### Statistical Analysis

Data were analyzed using ANOVA (*p*/0.05 = *). Duncan’s test was used to compare mean values.

## Results

### Plant Status and Growth

In plants treated with NaCl alone, a mortality of 6.6% was observed starting from 14 DAT, while in the biostimulated samples, no mortality was observed. Salt stress caused a decline in plant DW (dry weight) due to a reduction in leaf DW, while it did not affect the DW of roots and stem + lateral shoots (Table 1). No significant differences were found for the DW of the other parts. The Meg treatment countered the reduction in leaf DW, and the observed value for this parameter was not statically different from those found in the untreated controls (Table 1).

**Table 1:** Dry weight (DW) different parts and total DW of olive plants at 40 days after starting NaCl treatment (DAT).

	Roots (g)	Stem + lateral shoots (g)	Leaves (g)	Total (g)
Control	1.09^a^	1.33^a^	0.70^a^	3.12^a^
NaCl	1.17^a^	0.99^a^	0.39^b^	2.55^b^
NaCl + Meg	1.51^a^	0.98^a^	0.85^a^	3.34^a^

In each column, means followed by different letters are significantly different (*p* > 0.05).

### Leaves Gas Exchanges, Relative Water Content, and Chlorophyll Content

A decrease in Pn was observed in stressed samples when compared to the control, starting from 7 DAT prior to treatment with Meg. At 21 DAT, Meg treatment restored the values not statistically different to those of the control trees (Table 2). In general, the decrease in Pn was accompanied by a decrease in gs and an increase in Ci. Moreover, reduced values of gs were well correlated with E reduction (Table 2). The Meg treatment enhanced leaf RWC (relative water content; Table 3) and leaf chlorophyll content (Table 3) in NaCl + Meg trees, restoring values not statistically different to those exhibited by the control samples.

**Table 2:** Leaves gas exchanges at 7, 14, and 21 DAT.

	7 DAT	14 DAT	21 DAT
**Pn** **μmol (CO_2_) m^−2^ s^−1^**			
Control	1.52^a^	1.36^a^	1.77^a^
NaCl	-1.91^b^	0.28^b^	−1.87^b^
NaCl + Meg	-	0.20^b^	1.49^a^
**E** **mmol (H_2_O) m^−2^ s^−1^**			
Control	0.71^a^	1.14^a^	0.74^a^
NaCl	1.07^a^	0.72^b^	0.45^b^
NaCl + Meg	-	0.70^a^	0.67^a^
**gs** **mmol (H_2_O) m^−2^ s^−1^**			
Control	32.55^a^	53.87^a^	37.90^a^
NaCl	46.00^a^	39.17^a^	21.11^b^
NaCl + Meg	-	36.38^a^	37.01^a^
**Ci** **μmol mol^−1^**			
Control	367.30^a^	371.23^a^	315.89^a^
NaCl	459.59^a^	375.93^a^	425.28^b^
NaCl + Meg	-	388.18^a^	338.17^a^

In each column and for each parameter, mean values followed by different letters are significantly different (*p* > 0.05).

**Table 3:** Leaves relative water content (RWC) and leaf chlorophyll content (SPAD) at 7, 14, and 21 DAT.

RWC (%)				SPAD		
	7 DAT	14 DAT	21 DAT	7 DAT	14 DAT	21 DAT
Control	77.72^a^	70.53^a^	75.57^a^	71.34^a^	72.69^a^	78.40^a^
NaCl	77.80^a^	67.24^a^	60.28^b^	70.95^a^	65.76^b^	61.12^b^
NaCl + Meg	-	61.32^a^	74.86^a^	-	74.37^a^	79.92^a^

In each column, mean values followed by different letters are significantly different (*p* > 0.05).

### Na^+^, K^+^, Hydrogen Peroxide, and Malondialdehyde Contents in Olive Leaves

The sodium concentration in the leaves was significantly increased by the treatment with NaCl (+41%) when compared to the untreated controls (Table 4). Samples treated with NaCl in combination with Meg showed no significant differences in the Na^+^ content compared to samples grown with NaCl alone (Table 4). As for potassium, the K^+^ content found in NaCl-treated olive leaves was not significantly different from that shown by the control samples (Table 4). On the contrary, the olive stressed with NaCl and treated with Meg showed an increase in K^+^ content if compared to untreated controls (+44%) or samples treated only with NaCl (+33%; Table 4). As a result, biostimulated samples showed the highest K^+^/Na^+^ ratio, while those grown only with NaCl showed the lowest ratio (Table 4).

**Table 4:** Leaves Na^+^ and K^+^ content at 40 DAT.

	Na^+^ (mg g^−1^ dw)	K^+^ (mg g^−1^ dw)	K^+^/Na^+^
Control	0.39^b^	0.91^b^	2.33
NaCl	0.55^a^	0.98^b^	1.78
NaCl + Meg	0.47^ab^	1.30^a^	2.76

In each column, mean values followed by different letters are significantly different (*p* > 0.05).

**Figure 1:**
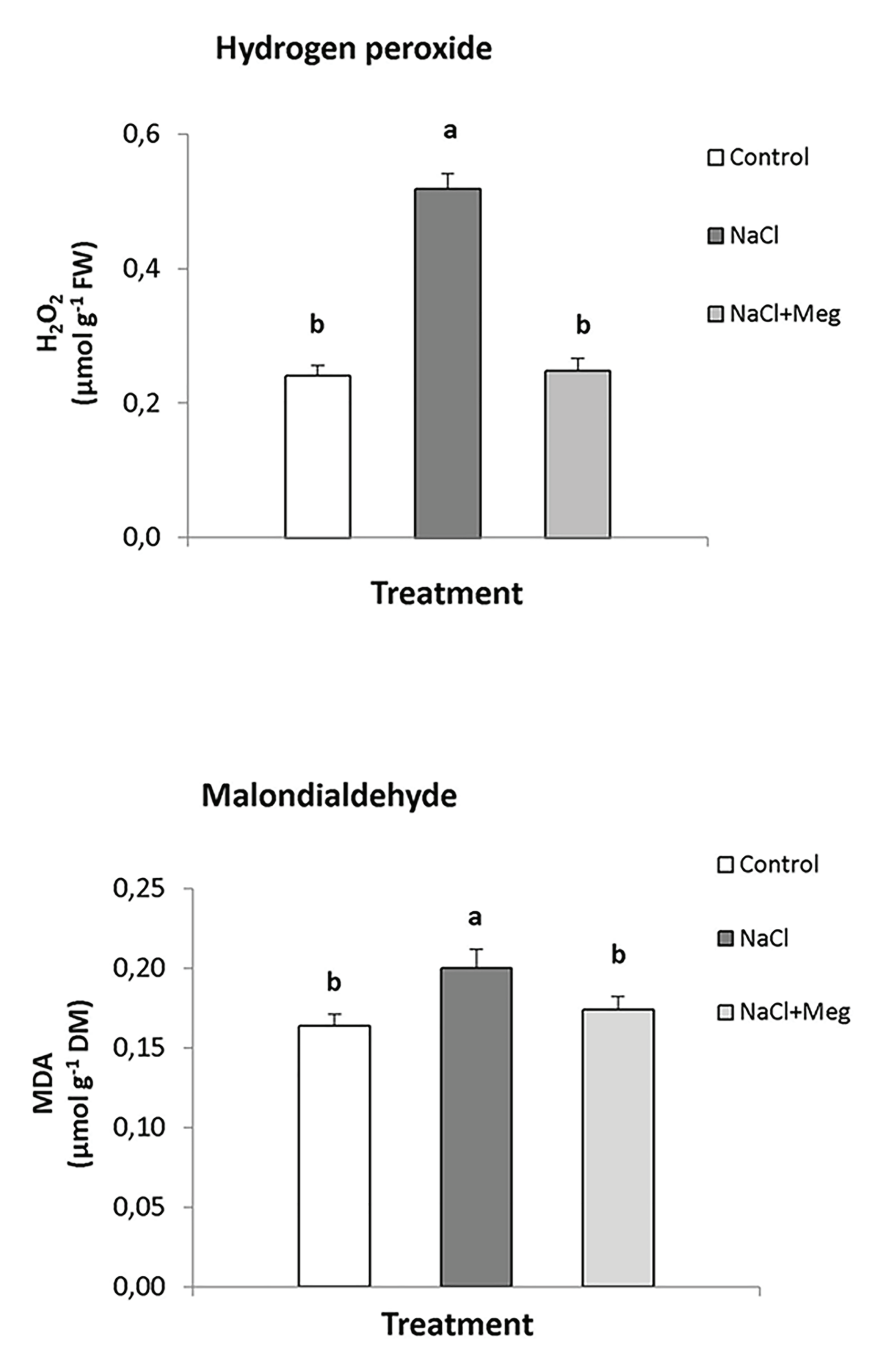
Hydrogen peroxide (H_2_O_2_) and malondialdehyde (MDA) contents found in olive leaves treated with NaCl alone or in combination with Megafol (Meg) and compared with those of untreated olive samples. For each treatment, means (±SD; *n* = 3) followed by different letters are significantly different (*p* > 0.05).

The top of Figure 1 shows the amounts of H_2_O_2_ and MDA in olive leaves of untreated samples (controls), plants treated with only NaCl, or plants treated with NaCl in combination with Meg. Based on the results, NaCl stress severely increased the content of H_2_O_2_ (+113%). Conversely, the plants treated with NaCl and biostimulated with Meg showed H_2_O_2_ levels not significantly different from those exhibited by the untreated controls (Figure 1).

Regarding MDA, the highest content of this lipid peroxidation product was found in NaCl-stressed samples, with values significantly higher than those found in the controls (+18%; bottom of Figure 1). In contrast, when the plants were exposed to saline stress and biostimulated with Meg, the amount of MDA accumulated by olive leaves returned to values, which were not statistically different from those shown by the control samples.

### Enzymatic Activities

**Figure 2:**
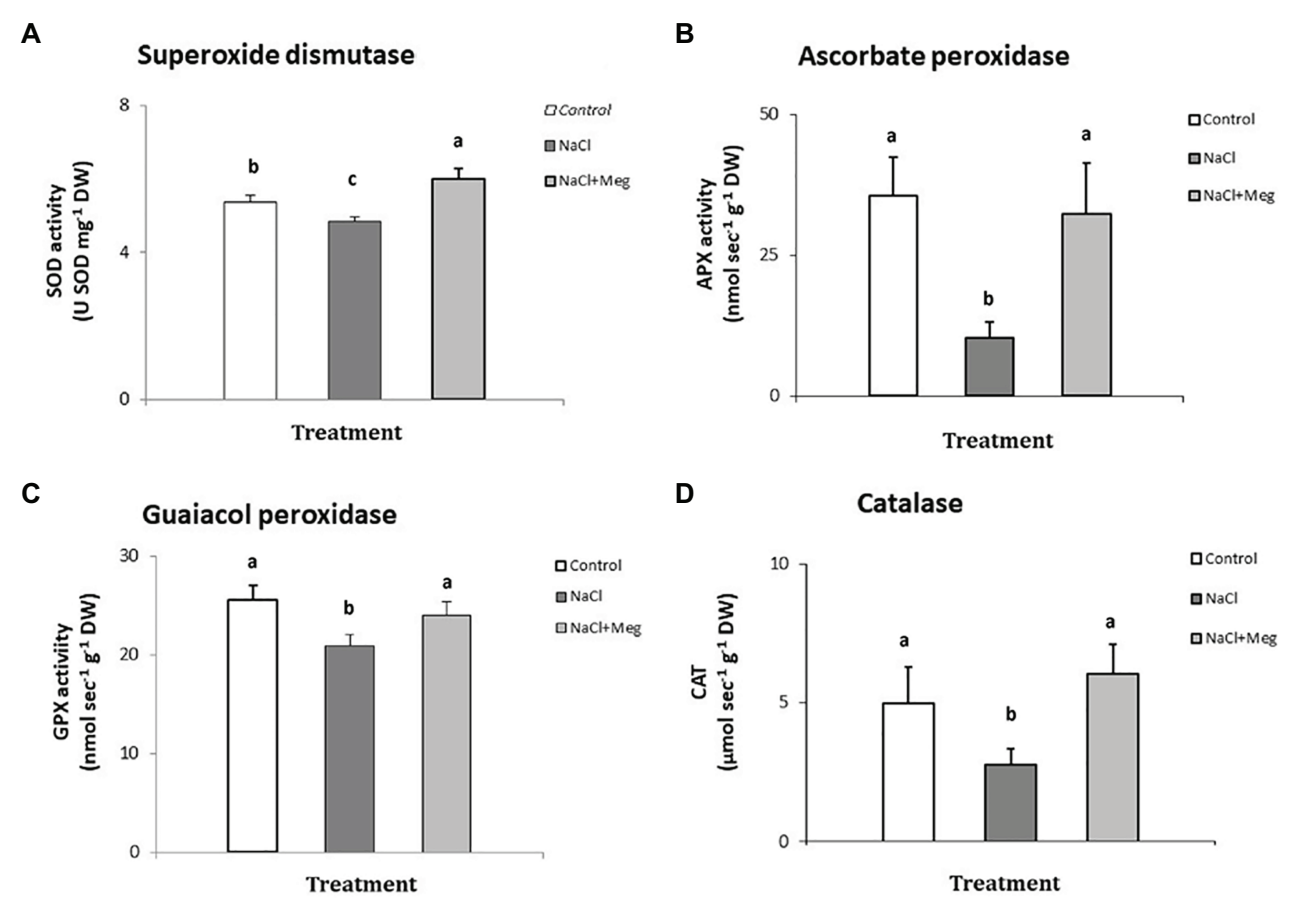
Superoxide dismutase (SOD) - (A), ascorbate peroxidase (APX) - (B), guaiacol peroxidase (GPX) - (C), and catalase (CAT) - (D) determined in samples of olive leaves treated with NaCl alone or in combination with Megafol and compared with those of untreated olive samples. For each treatment, means (±SD; *n* = 3) followed by different letters are significantly different (*p* > 0.05).

The activity of superoxide dismutase (Figure 2A) was significantly reduced in plants treated with NaCl (−10% compared to untreated olive samples). On the other hand, when the salt-stressed plants were biostimulated with Meg (NaCl + Meg), SOD activity showed the highest value, which was even higher than that of the untreated control (+11%).

Regarding APX (Figure 2B), NaCl strongly inhibited the activity of this enzyme in olive leaves (−68% compared to untreated plants). In contrast, when salt-stressed plants were biostimulated (NaCl + Meg), the APX activity recovered, reaching values not significantly different from those found for the untreated controls (Figure 2B).

In the guaiacol peroxidase assay (Figure 2C), salt-stressed olive leaves (NaCl) showed the lowest values of this enzymatic activity, which was statistically lower than those shown by the untreated control sample (−18%). In contrast, when NaCl-stressed plants were subjected to Meg treatment, the GPX activity recovered, showing values not statistically different from those found in the controls.

When investigating catalyis activity in NaCl-stressed plants, we observed a significant decrease in enzyme activity when compared to the untreated control samples (−20%; Figure 2D). On the contrary, when salt-stressed olive leaves were biostimulated with Meg, CAT activity recovered, reaching values not statistically different from those found in the untreated controls.

### Determination of Cytosolic Ca^2+^ in Olive Pollen Granules in the Presence of Meg

**Figure 3:**
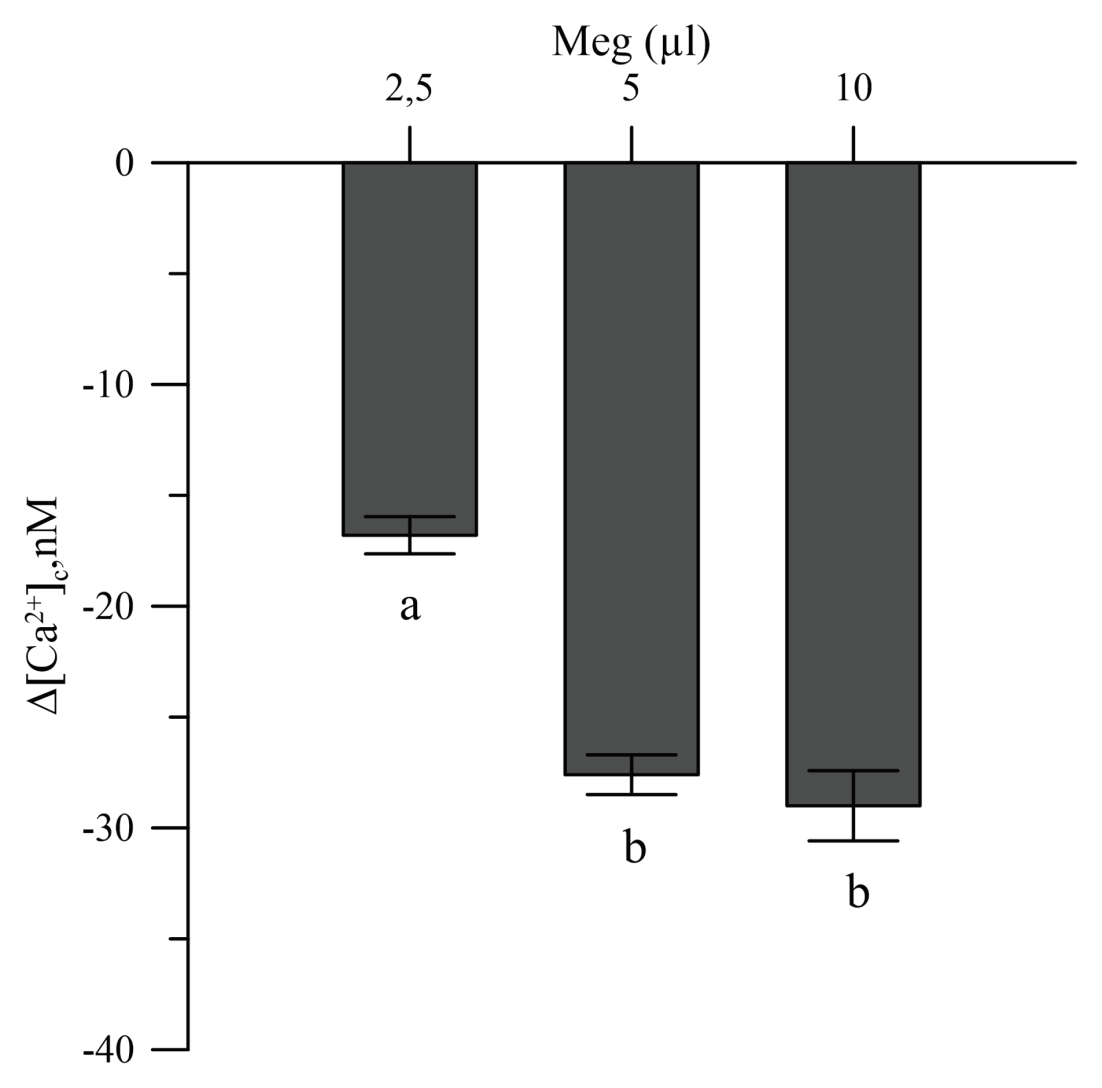
Effects of Meg on Ca^2+^-cytosolic acid of olive pollen grains. Values are expressed as means ± SEM from five independent tests.

The commercial biostimulant Meg exhibited a marked Ca^2+^ chelating activity in the cytosolic Ca^2+^ of the olive pollen grains labeled with the FURA 2 AM fluorescent probe. Meg showed high activity at a dose of 2.5 μl and became saturated above 5 μl (Figure 3).

### Effects of Meg on Cytosolic Ca^2+^ in Olive Pollen Grains Subjected to Oxidative Stress

**Figure 4:**
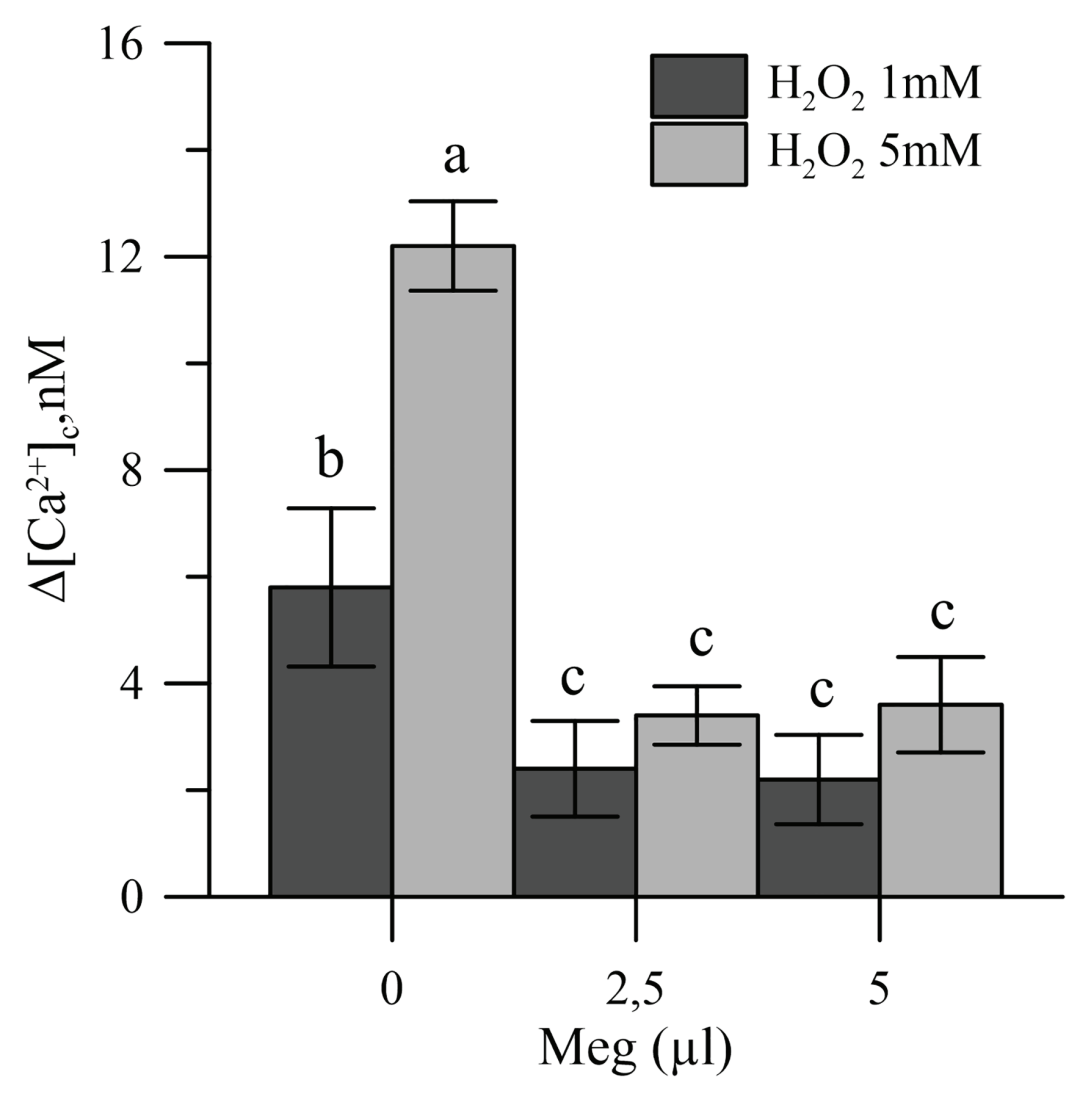
Effects of H_2_O_2_ (1 and 5 mM) on cytosolic Ca^2+^ of olive pollen in the presence and absence of Meg (2.5 and 5.0 ml). Values are expressed as means ± SEM from five independent tests.

Oxidative stress, induced *in vitro* in olive tree pollen with hydrogen peroxide (1 and 5 mM), caused an increase in the pollen cytosolic Ca^2+^ [(Ca^2+^)_cp_]. When the pollens were pre-treated with Meg, the effects of hydrogen peroxide disappeared (Figure 4).

## Discussion

The use of plant biostimulants is considered an innovative agronomic approach for its effectiveness in increasing crop productivity (Povero et al., 2016). Their stimulatory effects on flowering, nutrient use efficiency, plant productivity, and growth have been widely documented (Rouphael et al., 2015; Xu and Geelen, 2018). Besides, the use of this group of substances has been successfully proposed for improving crop resistance to a variety of harmful stresses (Calvo et al., 2014; Halpern et al., 2015; Bulgari et al., 2019).

In this context, the effectiveness of Meg, a commercial biostimulant, was tested on some physiological and biochemical traits of olive grown in hydroponic systems under saline stress. We also highlight the positive effect of this particular biostimulant on salt-stressed samples. To the best of our knowledge, this is the first study addressing a similar issue, although Meg is registered for treating olive crops.

We observed a reduction in leaf Pn in leaves under salt stress, which is in agreement with previous studies (Chartzoulakis, 2005; Mousavi et al., 2019; Regni et al., 2019) in which low and moderate salinity was associated with reduced CO_2_ assimilation rates. Generally, Pn reduction is accompanied by an increase in Ci that causes stomatal closure, with a consequent decrease in gs and E (Bacelar et al., 2007; Proietti et al., 2013). The increase in Ci suggests that the Pn reduction was mainly caused by non-stomatal effects and could be the result of specific or more generic damage to the photosystems under salt stress (Ben Ahmed et al., 2010; Singh and Reddy, 2011; Proietti et al., 2012). The Pn decrease in stressed samples caused a decline in plant growth mainly because of the reduction in leaf DW (Chartzoulakis, 2005; Karimi and Hasanpour, 2014). Karimi and Hasanpour (2014) also observed a reduction in leaf DW in salt-stressed plants due to the accumulation of toxic levels of salt, which caused premature senescence and abscission. The use of this biostimulant in stressed plants had a positive effect on Pn and, consequently, on plant DW, reducing the harmful effects of salt stress. The increase in DW provoked by Meg was caused both by a greater Pn and leaf area; Meg prompted a reduction in leaf fall and an improvement in the chlorophyll content (Tables 1 and 4).

Salinity can often influence the K^+^ content of plant cells in addition to Na^+^. The study of the dynamics of these minerals in plants is essential as some species can limit or control Na^+^ uptake as a defensive mechanism to better tolerate the stress (El Arroussi et al., 2018). On the other hand, plants can increase K^+^ uptake to cope with the harmful effects of salt stress (Desoky et al., 2018; El Arroussi et al., 2018). Indeed, it is known that increases in K^+^ contents can promote plant metabolism, growth and development, chlorophyll content and photosynthesis (Zrig et al., 2021). The results of this study showed that the olive treated with NaCl increased the Na^+^ content, while Meg was ineffective in influencing the amount of the cation adsorbed by the species (Table 4).

In contrast, samples grown in salinity and treated with Meg showed higher K^+^ contents than those found in the controls and samples treated with NaCl alone. In the specific case of salinity, such an effect is relevant because the increased K^+^ content can help plants to maintain growth, cell turgor, and gas exchange (Ahanger and Agarwal, 2016). Therefore, the results of this study are in line with the evidence mentioned above. In fact, the increased performance showed by samples grown in salinity but biostimulated with Meg may be related to the increase in K^+^.

One of the worst effects of salt toxicity is the overproduction of ROS which alter the cellular redox balance, thus generating oxidative stress (Del Buono et al., 2020). ROS negatively impact on cells (Asadi Karam et al., 2017; Boari et al., 2019) and are generated during photosynthesis and respiration because of intense electron flux (Liang et al., 2018). However, they can also act as signals involved in controlling decisive processes related to plant development and growth (Bulgari et al., 2019). When oxidative perturbations reach excessive levels, ROS accumulation can degrade proteins, alter the membrane stability, and damage the DNA (Del Buono et al., 2011; Nxele et al., 2017). As one of the main oxidizing species, H_2_O_2_, if accumulated under stress, can seriously compromise cell functionality (Lee et al., 2013).

The results of this study showed that saline stress increased the levels of H_2_O_2_ in olive leaves (Figure 1). In contrast, biostimulated plants showed H_2_O_2_ contents similar to those found in untreated control samples. To date, similar results have not been demonstrated for biostimulated olive crops irrigated with highly saline water, although for other species, reductions in the content of hydrogen peroxide, following treatment with biostimulants, have been reported. For example, *Solanum lycopersicum*, *Phaseolus vulgaris*, and *Cicer arietinum* grown under saline conditions and treated with biostimulants showed significantly reduced hydrogen peroxide contents (Ogweno et al., 2008; Abdel Latef et al., 2017; Martinez et al., 2018; Rady et al., 2019). This positive effect of Meg on the levels of H_2_O_2_ can also explain our results regarding the chlorophyll content, expressed as SPAD index (Table 4), as these pigments are the first targets of oxidants when cells are subjected to oxidative stress (Rady et al., 2018). Indeed, olive samples treated with NaCl alone accounted for a significant decrease in chlorophylls, indicating that the oxidant status caused by salt stress favored the oxidation of these pigments. These results are in line with those found for tomato plants exposed to drought stress and treated with Meg and a biostimulant obtained from the algae *Ascophyllum nodosum* (Petrozza et al., 2014; Goñi et al., 2018). In particular, biostimulated samples showed higher chlorophyll contents, reduced water losses, and lower levels of oxidized membranes when compared to un-biostimulated ones (Petrozza et al., 2014; Goñi et al., 2018).

Frequently, oxidative stress can damage plasma membranes (Desoky et al., 2019), and therefore, the MDA content in olive leaves was investigated. MDA is indicative of the integrity and functionality of membranes, being the main product of lipid peroxidation (Liang et al., 2018). Salt-stressed olive plants showed the highest levels of MDA (Figure 1), whereas plants grown under salinity and treated with Meg showed MDA contents comparable to those of the control samples. These findings are in line with those found for the hydrogen peroxide contents, further shedding light on the effectiveness of the biostimulant in improving the redox status of the cell. This aspect is crucial for plant survival and should be emphasized, as higher levels of MDA have been correlated with harmful electrolyte leakages and reduced cellular water content (Rady et al., 2019).

The observed beneficial effect exerted by Meg on olive plants, in terms of H_2_O_2_ and MDA contents, can be considered as a possible consequence of the increase in the activities of antioxidant enzymes induced by the biostimulant treatments. Plants can control ROS up to certain concentrations because of a wide range of ROS-removing enzymes (Colla et al., 2015). Among them, particularly important for plant protection are SOD, APX, GPX, and CAT (Colla et al., 2015; Rouphael et al., 2017). According to the previous studies, SOD is considered the first defense against oxidative stress and transforms the superoxide anion to O_2_ and H_2_O_2_ (Liang et al., 2018; Desoky et al., 2019); the H_2_O_2_ is then controlled by peroxidases and catalase (Ertani et al., 2013). Chloroplast APX is considered the main enzyme involved in H_2_O_2_ removal and acts by using ascorbic acid as a specific electron donor (Mimmo et al., 2015; Liang et al., 2018). Moreover, GPX, using guaiacol as electron donor, contributes to maintaining the redox status of the cell by removing hydrogen peroxide (Ertani et al., 2013). Finally, CAT, found exclusively in peroxisomes and glyoxysomes, removes H_2_O_2_ in leaves, mainly during light respiration (Willekens et al., 1994).

Regarding our results, we observed a general decrease in the activity of the antioxidant enzymes in olive grown only with NaCl (Figures 2A–D). The loss of activity was particularly severe in the case of APX, and this effect may be due to overaccumulation of oxidants and phytotoxicity of the salinity. Consequently, the drop in APX in NaCl-stressed samples, when compared to the controls and biostimulated olive plants, explains the accumulation of hydrogen peroxide, these enzymes are highly active in the removal of this oxidant (Ertani et al., 2013). Finally, the reduction in APX activity also explains the damages that presumably happened to the photosystems, as previously described, considering the pivotal role of these enzymes in protecting chloroplasts from oxidative damages (Liang et al., 2018).

The enzymes SOD, GPX, and CAT showed significant losses of activity in response to saline stress when compared to the untreated controls, which, however, were more contained than the loss of activity showed by APX. Overall, a general impairing effect on all antioxidant enzymes was observed following NaCl exposure, which can be related to the severity of the saline-induced stress, which overcame the antioxidant defensive enzymes, as reported previously. Bano et al. (2012) found that salinity, among other detrimental effects, strongly depressed the activities of SOD, CAT, and peroxidases, and this effect also depended on the cultivar tested. In general, olive is a crop moderately tolerant to salt (Regni et al., 2019), and its ability to cope with the toxicity caused by salinity can be cultivar-dependent (Rugini and Fedeli, 1990). In this context, we emphasize that a particular sensitivity to salt stress characterizes the olive cultivar investigated in this study.

Figure 2 reports the effect of Meg on the activity of antioxidant enzymes of olive samples grown under salt-stress. Regarding SOD, the biostimulant induced in the salt-stressed samples an enzymatic activity even higher than that shown by the untreated control samples. The induction of SOD is essential for protecting chloroplasts, since this enzyme removes the excess of anion superoxide in the cells (Zhao et al., 2007; Liang et al., 2018). Regarding the other enzymes investigated, Meg stimulated APX, GPX, and CAT in salt-stressed olive plants. The activities of these enzymes were not significantly different from those recorded for the untreated controls, suggesting that the right functionality of these hydrogen peroxide scavenger enzymes allowed the olive plants to remove this oxidant. Therefore, in the biostimulated samples, this beneficial effect resulted in the protection of the photosynthetic apparatus, chlorophylls, and lipids from oxidation and in an improved RWC of the plants. Most likely, treatment with Meg improved leaf RWC because of a greater water uptake by roots.

It should be noted that olive plants grown in conditions of high salinity accumulated H_2_O_2_ and MDA. In addition, saline stress reduced the activity of the antioxidant enzymes and the chlorophyll content. In contrast, Meg exerted a beneficial effect on this crop, allowing the biostimulated samples to recover in terms of the physiological and biochemical traits studied. For this reason, it was considered necessary to evaluate how Meg could have been involved in the biomolecular aspects of cells by monitoring the variation of cytosolic Ca^2+^ in olive pollen grains. The choice to study cytosolic Ca^2+^ was dictated by the wide involvement of this species as a molecular signal in processes essential for adjust cell functioning.

Furthermore, the maintenance of cytosolic Ca^2+^ at concentrations below 0.1 μM in the absence of stimuli is essential to preserve the cell’s perception systems.

Megafol showed chelating activity toward Ca^2+^, thus reducing the levels of the cytosolic ion and removing the effects of hydrogen peroxide in the Ca^2+^ cytosolic acid of the olive pollen grains (Figure 3).

The H_2_O_2_-dependent perturbations in the cytosolic Ca^2+^ in pollen grains could be the cause of abnormal molecular signals.

The correlation observed between cytosolic Ca^2+^ and germination highlighted in our previous work suggests that the germination capacity of pollen grains subjected to oxidative stress is linked to the maintenance of Ca^2+^ homeostasis (Del Pino et al., 2019a,b). The novelty of the treatment with Meg was the ability of the biostimulant to block the bioavailability of the cytosolic ion, thus avoiding unwanted transduction of signals in saline stress (Figure 4).

The ability of pollen from Meg-treated plants to tolerate oxidative stress is particularly important for agricultural productivity, based on multiple abiotic factors that can lead to excess ROS production.

Furthermore, the pollen of the olive tree, a well-known anemophilous plant, requires a large quantity of fertile pollen to obtain a good production (Koubouris et al., 2009).

## Conclusion

We could show that Meg can be used to increase the resistance of olive plants to severe salt stress conditions. Salinity strongly affected E, gs, and the photosynthetic activity of plants, probably damaging the photosystems because of the accumulation of H_2_O_2_ in the leaves. On the contrary, when the samples were biostimulated, some beneficial effect were found. In particular, the K^+^ content increased and the cell’s antioxidant machinery was generally induced. These effects indicate the protective mechanism of Meg action, which was also confirmed by agronomic and physiological data. In particular, biostimulated plants completely recovered the activity of some key antioxidant enzymes, thus avoiding the accumulation of hydrogen peroxide and lipid peroxidation. Finally, the biostimulant was able to interfere with cytosolic Ca^2+^, blocking its availability and maintaining its homeostasis. This additional protective mechanism of action was due to Meg’s chelating capacity towards Ca^2+^, which avoided perturbations to the ion homeostasis related to hydrogen peroxide increases. Such a beneficial effect prevented the formation of stress-mediated Ca^2+^ signals.

## Data Availability Statement

The original contributions presented in the study are included in the article/supplementary materials, further inquiries can be directed to the corresponding author/s.

## Author Contributions

Conceptualization and writing original draft: DB, LR, CP, and PP. Investigation: DB, LR, AP, MB, and PP. Methodology: DB, LR, AP, MB, CP, and PP. Supervision: DB, LR, and PP. All authors contributed to the article and approved the submitted version.

### Conflict of Interest

The authors declare that the research was conducted in the absence of any commercial or financial relationships that could be construed as a potential conflict of interest.
